# Global measure of satisfaction with psychosocial work conditions versus measures of specific aspects of psychosocial work conditions in explaining sickness absence

**DOI:** 10.1186/1471-2458-8-270

**Published:** 2008-08-01

**Authors:** Torsten Munch-Hansen, Joanna Wieclaw, Esben Agerbo, Niels Westergaard-Nielsen, Jens Peter Bonde

**Affiliations:** 1 Department of Occupational Medicine, Aarhus University Hospital, Aarhus, Denmark; 2 National Center for Register based Research, Aarhus University Hospital, Aarhus, Denmark; 3 School of Business, University of Aarhus, Denmark

## Abstract

**Background:**

Attempts to identify particular aspects of psychosocial work conditions as predictors of sickness absence remain inconclusive. A global measure has previously been suggested to be an efficient way to measure psychosocial work conditions in questionnaires. This paper investigates whether satisfaction with specific aspects of psychosocial work conditions explains sickness absence beyond its association with a global measure of psychosocial work conditions.

**Methods:**

The participants were 13,437 employees from 698 public service workplaces in Aarhus County, Denmark. 33 items from a questionnaire fell in groupings around six aspects of psychosocial work conditions: skill discretion, professionalism, management, decision authority, workload and cooperation. A global measure rating satisfaction with psychosocial work conditions on a scale from 0 to 10 was also included in the questionnaire. Individual ratings were aggregated to workplace scores. Analysis of variance and multiple linear regression was used to compare the average number of days of yearly sickness absence with different levels of satisfaction with six aspects of psychosocial work conditions. The covariates included were gender, age, occupation, size of workplace, contact to hospital, civil status and children below 13 living at home.

**Results:**

Dissatisfaction with each of the six aspects of psychosocial work conditions was associated with an increase in sickness absence. When all aspects were simultaneously included in the model, only skill discretion and professionalism were negatively associated with sickness absence. When a global measure of satisfaction with psychosocial work conditions was also included in the model none of the specific aspects showed a statistically significant association with sickness absence.

**Conclusion:**

Low global satisfaction with psychosocial work conditions is associated with increased levels of sickness absence. Including specific aspects of psychosocial work conditions in the model does not provide further information regarding the nature of this association.

## Background

Many studies have attempted to establish the association between specific aspects of psychosocial work conditions and sickness absence. [1-10] Most previous studies examined Karasek's demands-, control- (and support-) model[11] and Siegrist's effort-reward imbalances model[12] as predictors of sickness absence or were inspired by these models.[13] Other aspects have also been discussed and applied (organizational justice, leadership). Findings are most consistent regarding the association between low decision authority and increased levels of sickness absence.[1,4,5,7,10,14] Evidence concerning the impact of other aspects of psychosocial work conditions is limited and inconsistent [15].

In a previous study we examined the association between a global measure of psychosocial work conditions and sickness absence. We found a positive correlation between the level of satisfaction with psychosocial work conditions and the amount of sickness absence across different occupations and types of work places. A global measure is a parsimonious way of measuring psychosocial work conditions, assuming that the overall concept of psychosocial work conditions is conceived in a more homogenous way across heterogenous populations than separate aspects of psychosocial work conditions.

The purpose of the present study was to investigate the relationship between specific aspects of psychosocial work conditions and sickness absence in a large sample of public services employees. Additionally it examines whether specific aspects of psychosocial work conditions explain sickness absence beyond the association found regarding the global measure. The study includes aspects of psychosocial work conditions evaluating professionalism and management that are not commonly included in studies regarding psychosocial work conditions.

Two specific questions raised are i) do specific aspects of psychosocial work conditions function as independent predictors of sickness absence beyond a one-dimensional global measure of satisfaction with psychosocial work conditions? And ii) does the character of the association between psychosocial work conditions and sickness absence vary across different occupations?

## Methods

### Study population

From April 2002 through April 2005 Aarhus County in Denmark conducted a general survey of the psychosocial work conditions among all employees, with the intent to use the results to improve psychosocial work conditions. [16]. The surveys were performed anonymously at each workplace unit. A workplace unit was defined as the lowest organizational level up to the first level of Management. The size of the workplace units ranged from 4 to 120 employees. The surveys were organized by the Department of Quality, Aarhus County.

The study population included 13,437 employees from 698 different workplaces in Aarhus County who received a questionnaire in one of the surveys (Additional file 1). The surveys were spread across all seasons except the months of summer vacation (July and August). The overall response rate for the workplace-units included in the study was 81.0%. The use of these data and of sickness absence and register data for the employees for this study was approved by the Danish Data Protection Agency. No human subjects committee was addressed because the study was register based and thus no contacts were made to the participants.

### Measurement of psychosocial work conditions

The surveys of psychosocial work conditions were performed using a questionnaire containing 40 specific items and a global question. The 40 items were statements and the respondents were asked to assess to what degree the statements applied to their perception of the psychosocial work conditions on a four-point scale ranging from "yes, very much" to "no, not at all". The 40 items were chosen from a pilot questionnaire tested in 2001 including 60 items answered by 943 employees at 47 workplace units. The items were inspired by questionnaires used in similar studies [13,17,18]. 33 of the 40 items were selected for this study. 7 questions were excluded because they were referring to the experience of certain staff policies not present at all workplaces or because they were too similar to the general global question. The global question was: How satisfied are you, all in all, with the psychosocial work conditions at your workplace?" The responses were rated on a scale from 0 (unacceptable) to 10 (exceptional).

The 33 items were clustered around 6 different aspects of psychosocial work conditions: skill discretion, professionalism, management, decision authority, workload and cooperation. The internal consistency of the questionnaire was tested by measuring the Cronbach's alpha values for the aspects of the study. Additional file 2 presents the scales. The Cronbach's alpha-values for the 6 scales are listed.

In a sample of 369 social care workers the convergent validity of three of the scales [19] of the questionnaire was tested by comparing them with similar scales from the Copenhagen Psychosocial Questionnaire [13]. The correlation coefficients were 0.81 for the management scale, 0.61 for the decision authority scale and 0.40 for the workload scale.

The discriminant validity of the scales (their ability to measure genuinely different aspects of psychosocial work conditions) can be evaluated by a simple correlation matrix (Table 1).

**Table 1:** Correlations between six aspects of psychosocial work conditions and a global measure (Pearsons Correlation Coefficient)

	**Management**	**Cooperation**	**Professionalism**	**Skill Discretion**	**Workload**	**Decision Authority**	**Global measure**
**Management**	1.00	0.57	0.64	0.46	0.33	0.67	0.64
**Cooperation**		1.00	0.58	0.41	0.29	0.51	0.60
**Professionalism**			1.00	0.48	0.40	0.61	0.62
**Skill discretion**				1.00	0.34	0.50	0.45
**Workload**					1.00	0.41	0.40
**Decision Authority**						1.00	0.59
**Global measure**							1.00

Four of the scales were closer correlated than the others (management, cooperation, professionalism and decision authority) with coefficients between 0.51 and 0.67. The same four aspects were also closer correlated to the global measure than skill discretion and workload. Workload and skill discretion were least correlated to the other aspects (Pearson's correlation coefficients between 0.29 and 0.50).

The content validity was evaluated by two occupational psychologists. They were asked to place the original 40 items under headings with aspects commonly regarded as key aspects of psychosocial work conditions (decision authority, predictability, social support, demands/workload, skill discretion and rewards). The questionnaire was found to include measures of all of these aspects.

### Measures of sickness absence

Data regarding sickness absence were obtained from the salaries administrative system of the County of Aarhus. Reporting of sickness absence is compulsory and necessary to obtain wage reimbursement. The data are produced by each workplace and submitted to the administrative system each week. The data contain a record for each day of absence for every employee. Only the instances explicitly coded with illness as the reason for absence were included, thus excluding maternity leave and other reasons for absence. Data regarding sickness absence could only be linked to data regarding psychosocial work conditions at the workplace level, as the latter were not available at the individual level. The outcome measure was the number of days of sickness absence through one year for each employee. The number of spells of sickness absence was also examined. The distribution of spells was similar to the distribution of the number of days and the association to psychosocial work conditions was also similar. Choosing spells of sickness absence as our outcome measure would thus have lead to the same conclusions. The time period used was 6 months before and 6 months after the time of measurement of psychosocial work conditions. As only the month of measurement of satisfaction with psychosocial work conditions was known the 15^th ^was chosen as the start date of measurement of sickness absence.

### Covariates

The covariates included in the adjusted analyses were: gender (female or male); age (continuous); occupation (the 12 most common occupations in the County of Aarhus and a residual category); the size of the workplace unit (30 employees or less; more than 30 employees); marital status (living with a partner; living alone); children (any children below 13 years; no children below 13 years); and registered illness during the year of measurement of sickness absence (any contact to hospital; no contact to hospital).

### Statistical analyses

The responses to the items of each aspect were added using equal weights and scaled from 0 to 100. The global measure was also transformed to a 0 to 100 scale. The aggregated workplace unit scores from each of the 698 workplace-units were assigned to each employee for all of the aspects.

The aggregate scores where divided into septiles. A low level of satisfaction was defined as the lowest septile, medium satisfaction as the 5 middle septiles and high satisfaction as the highest septile. The levels were defined to satisfy a trade off between the size of the groups and the magnitude of contrast between the groups. No absolute cut off point could be used across the 6 aspects as the distributions cover different spans of the 0 to 100-scale.

Analyses were performed using the workplace unit scores on the 6 aspects of psychosocial work conditions as predictors of the individual number of days of sickness absence throughout a one-year period. In the first step of the analyses we compared the number of sickness absence days during a year for the three levels of satisfaction for each aspect and the global measure. Analysis of variance was used to test differences between mean values of sickness absence across the three levels of the scores. In a second step we used multiple linear regression to estimate the effect of a one-unit increase in the satisfaction with psychosocial work conditions on sickness absence.

Estimates were calculated including the covariates with and without control for the other aspects in order to investigate how much the individual aspects contributed to prediction of sickness absence.

**Figure 1:**
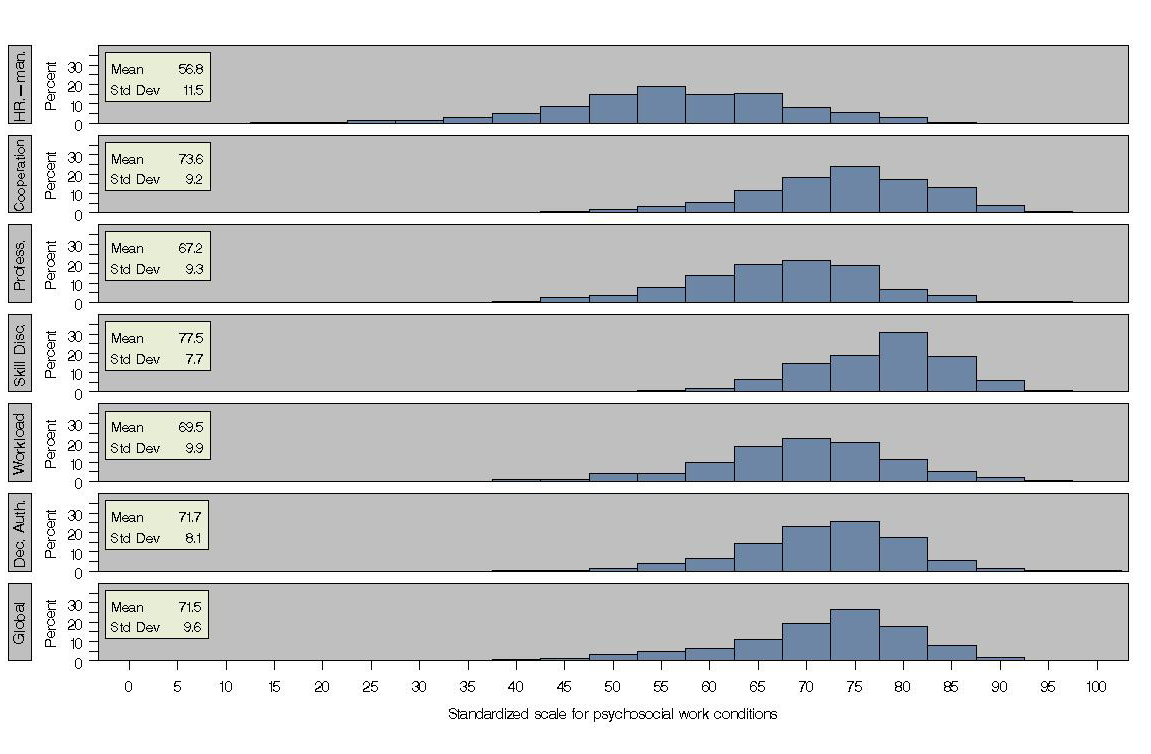
Distribution of 6 aspects and a global measure of psychosocial work conditions.

The SAS 9.13 statistical package was used for all statistical analyses. Analysis of variance and multiple linear regression was performed using the MIXED procedure with the repeated option account for the workplace clustered nature of the data on satisfaction with psychosocial work conditions. All covariates were included in the model regardless of their contribution to the explanation of sickness absence variation. The distributions of the scores on the 6 aspects and of the global measure of psychosocial work conditions are presented in figure 1.

Distribution of 6 aspects and a global measure of psychosocial work conditions

## Results

Table 2 shows the association between the 6 aspects of psychosocial work conditions and sickness absence.

**Table 2:** Days of sickness absence during one year according to level of satisfaction with psychosocial work conditions.

	Levels of satisfaction with psychosocial work conditions			
	Lowest septile	5 middle septiles	Highest septile	Regression coefficients

Aspect of psychosocial work conditions	Days of sickness absence during one year			

Management				
Adjusted mean^1 ^(95% CI)	14.3 (12.8 ; 15.9)	12.5 (11.6 ; 13.4)	11.6 (9.9 ; 13.2)	-0.08 (-0.13 ; -0.03)
Adjusted mean^2 ^(95% CI)	13.5 (11.4 ; 15.5)	13.1 (11.7 ; 14.5)	13.1 (11.1 ; 15.1)	-0.05 (-0.14 ; 0.04)
Adjusted mean^3 ^(95% CI)	11.9 (10.1 ; 13.8)	12.7 (11.8 ; 13.6)	13.4 (11.7 ; 15.2)	0.05 (-0.03 ; 0.13)
Cooperation				
Adjusted mean^1 ^(95% CI)	14.3 (12.7 ; 15.9)	12.5 (11.6 ; 13.4)	11.5 (9.9 ; 13.2)	-0.08 (-0.15 ; -0.02)
Adjusted mean^2 ^(95% CI)	13.8 (11.9 ; 15.6)	13.1 (11.8 ; 14.3)	12.9 (11.0 ; 14.8)	0.01 (-0.08 ; 0.09)
Adjusted mean^3 ^(95% CI)	12.3 (10.5 ; 14.1)	12.6 (11.7 ; 13.5)	13.3 (11.5 ; 15.1)	0.07 (-0.02 ; 0.16)
Professionalism				
Adjusted mean^1 ^(95% CI)	15.1 (13.5 ; 16.6)	12.4 (11.5 ; 13.3)	10.9 (9.3 ; 12.5)	-0.12 (-0.18 ; -0.06)
Adjusted mean^2 ^(95% CI)	15.2 (13.2 ; 17.3)	13.1 (11.5 ; 14.6)	11.4 (9.4 ; 13.5)	-0.11 (-0.22 ; 0.00)
Adjusted mean^3 ^(95% CI)	13.0 (11.0 ; 15.0)	12.6 (11.7 ; 13.5)	12.6 (10.7; 14.4)	0.04 (-0.06 ; 0.15)
Skill discretion				
Adjusted mean^1 ^(95% CI)	14.3 (12.7 ; 15.9)	12.5 (11.6 ; 13.4)	11.4 (9.7 ; 13.0)	-0.15 (-0.23 ; -0.08)
Adjusted mean^2 ^(95% CI)	14.2 (12.4 ; 16.1)	13.0 (11.7 ; 14.3)	12.5 (10.7 ; 14.3)	-0.11 (-0.21 ; -0.02)
Adjusted mean^3 ^(95% CI)	13.2 (11.6 ; 14.9)	12.5 (11.6 ; 13,4)	12.6 (10.9 ; 14.3)	-0.05 (-0.14 ; 0.03)
Workload				
Adjusted mean^1 ^(95% CI)	13.4 (11.5 ; 15.3)	12.8 (11.6 ; 14.1)	13.5 (11.7 ; 15.2)	-0.04 (-0.10 ; 0.02)
Adjusted mean^2 ^(95% CI)	13.5 (9.6 ; 17.4)	15.1 (12.2 ; 18.0)	12.3 (8.5 ; 16.0)	0.01 (-0.06 ; 0.08)
Adjusted mean^3 ^(95% CI)	12.3 (10.6 ; 14.0)	12.5 (11.7 ; 13.4)	13.5 (11.8 ; 15.1)	0.05 (-0.01 ; 0.11)
Decision Authority				
Adjusted mean^1 ^(95% CI)	14.2 (12.7 ; 15.8)	12.4 (11.5 ; 13.4)	11.6 (10.0 ; 13.3)	-0.10 (-0.17 ; -0.03)
Adjusted mean^2 ^(95% CI)	12.7 (10.7 ; 14.8)	13.2 (11.7 ; 14.8)	13.8 (11.7 ; 14.8)	0.11 (-0.04 ; 0.25)
Adjusted mean^3^(95% CI)	11.3 (9.4 ; 13.2)	12.7 (11.8 ; 13.7)	14.0 (12.2 ; 15.9)	0.14 (0.03 ; 0.25)
Global measure				
Unadjusted mean (95% CI)	16.3 (14.7 ; 17.9)	11.8 (11.1 ; 12.5)	11.2 (9.6 ; 12.8)	-0.17 (-0.23 ; -0.11)
Adjusted mean^1 ^(95% CI)	16.2 (14.7 ; 17.8)	12.1 (11.2 ; 13.0)	11.0 (9.4 ; 12.6)	-0.16 (-0.21 ; -0.10)

Bold types indicate that the estimate is statistically significant. For the three levels of satisfaction with psychosocial work conditions bold types indicate a statistically significant difference compared to the previous (lower) level.

1: Adjustment for gender, age, occupation, size of workplace, marital status, children below 13 years living at home and contact to hospital.

2: Adjustment for gender, age, occupation, size of workplace, marital status, children below 13 years living at home and contact to hospital and each of the other 5 aspects.

3: Adjustment for gender, age, occupation, size of workplace, marital status, children below 13 years living at home and contact to hospital and a global measure of psychosocial work conditions.

The results from the first step of the analyses showed a statistically significant association between increasing satisfaction and decreasing sickness absence for all aspects except workload. For the 5 other components the decrease in sickness absence was statistically significant between the lowest and the medium level of satisfaction with psychosocial work conditions while the difference between the medium and the highest level was not statistically significant. Skill discretion, professionalism and decision authority were most closely associated with sickness absence. A 10 points increase in satisfaction with psychosocial work conditions on each of these 3 aspects was associated with a 1 – 1.5 days decrease in sickness absence per year.

In the second step of the analyses, when all 6 aspects were included in the model only the skill discretion-aspect was statistically significantly associated with sickness absence (-0.11 (95% CI: -0.21 ; -0.02)). The professionalism aspect had an equally high but not statistically significant regression coefficient (-0.11 (95% CI: -0.22 ; 0.00)).

In a third step we tested whether any of the aspects could independently predict sickness absence when a global measure of satisfaction with psychosocial work conditions was also included in the model. Only decision authority had a statistically significant, independent association with sickness absence (0.14 (95% CI: 0.03 ; 0.25)) and the effect was in the opposite direction than expected. Regression coefficients were positive (but non-significant) for all other aspects except skill discretion. The global measure was consistently negatively associated with sickness absence regardless of which of the 6 aspects were included in the model and it also had the strongest explanatory power in itself (-0.16 (-0.21 ; -0.10).

The effect of poor satisfaction with psychosocial work conditions on sickness absence could not be further explained by including specific aspects of psychosocial work conditions in the model. The overall results from the model with all 6 aspects did not apply generally across all occupations included in the study. For some occupations other aspects than skill discretion or professionalism were closer associated with sickness absence. None of these associations were statistically significant when all 6 aspects were included in the model. For most occupations none of the aspects were associated with sickness absence (results not shown).

## Discussion

We found that all aspects of psychosocial work conditions included in the study, except workload, were individually negatively associated with sickness absence. The amount of sickness absence was notably lower in the group with medium satisfaction with psychosocial work conditions than in the most dissatisfied group. When all aspects were included simultaneously in the model, skill discretion and professionalism showed the strongest association with sickness absence. The meaning of work, development opportunities and the deployment of personal and professional resources, which are inherent in the skill discretion aspect thus provide the best target for improving psychosocial work conditions in order to reduce sickness absence. Skill discretion was not highly correlated with the global measure, thus rendering an independent explanatory power on sickness absence possible.

The negative association between professionalism and sickness absence (regression coefficient -0.11) was not statistically significant. Professionalism was more closely correlated to the global measure than skill discretion.

When a global measure of psychosocial work conditions was included in the model, only one of the six aspects of psychosocial work conditions, namely decision authority, was associated with sickness absence. Surprisingly, an increase in decision authority was associated with increased sickness absence. The reason for this independent explanatory contribution could be that decision authority is not always contributing to enhance the psychosocial work conditions. Increased decision authority means increased responsibility and new ways of working can lead to a blurring of work and private life. The result could also be an artifact due to inclusion of overlapping variables in the analysis.

Specific aspects of psychosocial work conditions have not explained sickness absence beyond its negative association with the global measure found in our previous study.

Most previous studies have implicitly assumed that psychosocial work conditions can be divided into a number of clearly distinguishable aspects that can be consequently identified as latent variables across heterogeneous populations and thus measured with the use of generic questionnaires.

Typically studies took offset in Karasek's demands, control (and support) model [11] or Siegrist's effort-reward imbalances model [12] to identify predictors of sickness absence. These studies suggested an association between low decision authority and increased sickness absence [1,4,5,7,10,14], whereas evidence of the impact of other aspects of psychosocial work conditions remains limited and inconsistent. In these studies the associations between a global measure of psychosocial work conditions and sickness absence was not examined. Therefore it remains unclear whether inclusion of a global measure in these studies would have eliminated the associations found between specific dimensions of psychosocial work conditions and sickness absence.

The use of a global measure provides the opportunity to examine the explanatory power of specific aspects as compared to the global measure. This study suggested that psychosocial work conditions may be more of a one dimensional concept than previous studies have hypothesized, because the global measure explained more of the negative association between psychosocial work conditions and sickness absence than any of the aspects included in this study.

If the specific aspects of psychosocial work conditions do not have any independent explanatory power when a global measure is included, then the aspects do not explain the association beyond the association found between sickness absence and the global measure.

When only a limited number of items or aspects are included in a study, they could all account for the effect of the common latent aspect "psychosocial work conditions" and could lead to false conclusions. Furthermore, even when the association between a global measure of psychosocial work conditions and sickness absence is consistent across subpopulations, the combination of aspects of psychosocial work conditions responsible for this association may be different across subpopulations. The inconsistent results of earlier studies suggest that this indeed may be the case.

Subpopulations are typically divided according to gender, occupation or type of institution. It also remains unclear whether specific dimensions of psychosocial work conditions are associated with sickness absence in the same manner across all of these subpopulations. Particular characteristics of the workplace amounting to a workplace "culture" may be more important for the association than e.g. different occupational categories.

The strengths of this study are that it is based on a large and heterogeneous study sample and in the independent measurement of exposure and outcome. Our data on sickness absence are highly reliable, as the Danish legislation requires accurate registration for reimbursement of expenses. Equally the response rate in the surveys of psychosocial work conditions was high (81.0%). The validity of the 6 scales used in this study was evaluated through measurements of internal consistency, convergent, discriminant and content validity. On these criteria they performed adequately. The inclusion of the global measure of psychosocial work conditions allowed for further testing of the discriminant validity. However, the study also has some limitations: important predicting variables such as physical demands[6,20], shift work [21-25], individual lifestyle, attitudes regarding sickness absence and physical constitution were not included. These variables may be related to both proneness to report low satisfaction with psychosocial work conditions and an individual susceptibility to sickness absence.

Individual data regarding sickness absence could only be linked to workplace levels of psychosocial work conditions. This is both a limitation, possibly responsible for weakening the association found in the study but also a strength as the use of workplace unit scores may minimize the effect of individual characteristics not related to the workplace as the effect of these variables may be expected to be randomly distributed across workplaces in our study sample.

Our design provides a better background to believe that our results represent an association truly related to satisfaction with psychosocial work conditions as it avoids the triviality trap of personal characteristics associated to both proneness to sickness absence and reporting of low satisfaction with psychosocial work conditions[26].

## Conclusion

We have earlier found a strong and consistent association between general satisfaction with psychosocial work conditions and sickness absence. The present study indicates that this association is not further explained by including measures of specific aspects of psychosocial work conditions as management, cooperation, skill discretion, professionalism, work load and decision authority.

Instead it is a general dissatisfaction with psychosocial work conditions that is associated to sickness absence rather than a specific combination of different aspects of the psychosocial work conditions. No particular aspects can be identified as associated clearly to sickness absence neither in the total study population nor in occupational subgroups.

A general recommendation for workplaces with poor psychosocial work conditions would be to focus particularly on the items and aspects with low scores in their own local survey results instead of pointing to specific aspects as being generally associated to sickness absence across all subpopulations. It can also be recommended to focus particularly on workplaces with low satisfaction with psychosocial work conditions because the difference in amount of sickness absence is most notable between the groups of workplaces with low and medium levels of satisfaction.

Across large and heterogenous populations associations between psychosocial work conditions and sickness absence we find it appropriate to use a single global question as a screening to spot workplaces with particular problems regarding psychosocial work conditions. In a forthcoming study concerning interventions to improve psychosocial work conditions we argue, that only at workplaces with poor psychosocial work conditions (the lowest septile) there is an effect on sickness absence of intervening to improve psychosocial work conditions. At the workplaces spotted through the screening it will make sense to make a more detailed survey of psychosocial work conditions to clarify the aspects of psychosocial work conditions most in need for improvement and as indicators for measurement of the effect of subsequent interventions.

## Competing interests

The authors declare that they have no competing interests.

## Authors' contributions

TM was the main author of the manuscript and has contributed to all phases of it.  JW and JPB contributed substantially to the conception, design, analysis and interpretation of data and the drafting and revision of the manuscript.  EA and NW contributed substantially to the analysis and interpretation of data and to the revision of the manuscript. All authors read and approved the final manuscript.

## Pre-publication history

The pre-publication history for this paper can be accessed here:



## Supplementary Material

Additional file 1 Characteristics of 13,437 participants. Click here for file

Additional file 2 Six aspects of psychosocial work conditions and the items used to measure them (Cronbachs α values for the scales). Click here for file
