# Supplementary material for: Global measure of satisfaction with psychosocial work conditions versus measures of specific aspects of psychosocial work conditions in explaining sickness absence
Source: BMC Public Health. 2008 Aug 1;8:270. doi: 10.1186/1471-2458-8-270 (PMC2518561; doi:10.1186/1471-2458-8-270)
Supplement: Additional file 1 — Characteristics of 13,437 participants. [file 1471-2458-8-270-S1.doc]

# Table 1 Characteristics of 13,437 participants

|  |  | **N (% of total)** |
| --- | --- | --- |
| Total |  | 13,437 (100.0) |
| Gender | Female | 10,568 (78.6) |
|  | Male | 2,869 (21.4) |
| Age | Less than 30 | 1,452 (10.8) |
|  | 30-39 | 3,556 (26.5) |
|  | 40-49 | 4,144 (30.8) |
|  | 50-59 | 3,634 (27.0) |
|  | 60 or more | 651 (4.8) |
| Type of workplace | Hospitals | 7,704 (57.3) |
|  | Educational or rehabilitation institutions | 1,906 (14.2) |
|  | Special care institutions, children and adolescents | 1,822 (13.6) |
|  | Psychiatric institutions | 1,430 (10.6) |
|  | Road and nature care | 334 (2.5) |
|  | Administration | 241 (1.8) |
| Occupation | Nurses | 3,669 (27.3) |
|  | Nursing assistants | 1,474 (11.0) |
|  | Office clerks | 1,294 (9.6) |
|  | Unskilled service workers | 1,032 (7.7) |
|  | Other health professionals | 1,015 (7.6) |
|  | Social care workers | 1,013 (7.5) |
|  | Secondary school teachers | 963 (7.2) |
|  | Academics | 668 (5.0) |
|  | Primary school teachers | 573 (4.3) |
|  | Physicians | 486 (3.6) |
|  | Craftsmen | 294 (2.2) |
|  | Technical assistants | 192 (1.4) |
|  | Other | 764 (5.7) |
| Size of workplace | Up to 30 employees | 8,744 (65.1) |
|  | More than 30 employees | 4,693 (34.9) |
| Contact to hospital | No | 8,299 (61.8) |
|  | Yes | 5,138 (38.2) |
| Civil status | Living with partner | 7,937 (59.1) |
|  | Living alone | 5,500 (40.9) |
| Children below 13 years | No | 8,722 (64.9) |
|  | Yes | 4,715 (35.1) |
