# Supplementary material for: Global measure of satisfaction with psychosocial work conditions versus measures of specific aspects of psychosocial work conditions in explaining sickness absence
Source: BMC Public Health. 2008 Aug 1;8:270. doi: 10.1186/1471-2458-8-270 (PMC2518561; doi:10.1186/1471-2458-8-270)
Supplement: Additional file 2 — Six aspects of psychosocial work conditions and the items used to measure them (Cronbachs α values for the scales). [file 1471-2458-8-270-S2.doc]

**Table 2 Six aspects of psychosocial work conditions and the items used to measure them (Cronbachs α** values for the scales)

| **Management (Cronbachs α=0.91)** | **Skill discretion (Cronbachs α=0.84)** |
| --- | --- |
| - The management is motivating and inspiring - The management swithces appropriately between listening and talking - The management has a good grasp of the workplace - It pays off to talk to the management about difficulties in my work - Conflicts are solved appropriately at the workplace - The manager is capable of decisive action - The management gives me favorable and critical comments in a way that motivates me to improve my work - My effort is appreciated | - I am satisfied with the challenges I get through my work - The work gives me the opportunity to exercise my personal and professional skills - My job is exciting - My work is appropriately varied - My workplace gives me the opportunity for personal and professional development |
| **Cooperation (Cronbachs α=0.85)** | **Workload (Cronbachs α=0.67)** |
| - I feel comfortable with the rhetoric and the social conventions at the workplace - I find that the workplace is characterized by a cooperative spirit - We treat each other as equals and with respect - We support each other in difficult situations at work - At the workplace we give each other favorable and critical comments in a way that motivates me to improve my work - In my experience there is equality of opportunity at my workplace (across e.g. age, gender or ethnicity) - Everybody can say his opinion freely at my workplace | - I can combine the demands at work with a sound private life - I am satisfied with my daily amount of work |
| **Professionalism (Cronbachs α=0.86)** | **Decision Authority (Cronbachs α=0.69)** |
| - The products of my workplace are of high quality - There is agreement between our daily work and our values and goals - In my experience there is agreement about the values and goals of the workplace - At the workplace we are preoccupied by improving the quality of the work - Time and energy are deployed for the right purposes - My workplace enjoys respect among users and collaborators - If something doesn’t work it is addressed appropriately | - I find that the employees are involved appropriately when decisions are made - I am satisfied with my opportunity to influence the planning of my work - I have the information I need to perform my work - I know what is expected from my work |
